# Metaproteomics of complex microbial communities in biogas plants

**DOI:** 10.1111/1751-7915.12276

**Published:** 2015-04-15

**Authors:** Robert Heyer, Fabian Kohrs, Udo Reichl, Dirk Benndorf

**Affiliations:** 1 Bioprocess Engineering, Otto von Guericke University MagdeburgUniversitätsplatz 2, Magdeburg, 39106, Germany; 2 Max Planck Institute for Dynamics of Complex Technical SystemsSandtorstr. 1, Magdeburg, 39106, Germany

## Abstract

Production of biogas from agricultural biomass or organic wastes is an important source of renewable energy. Although thousands of biogas plants (BGPs) are operating in Germany, there is still a significant potential to improve yields, e.g. from fibrous substrates. In addition, process stability should be optimized. Besides evaluating technical measures, improving our understanding of microbial communities involved into the biogas process is considered as key issue to achieve both goals. Microscopic and genetic approaches to analyse community composition provide valuable experimental data, but fail to detect presence of enzymes and overall metabolic activity of microbial communities. Therefore, metaproteomics can significantly contribute to elucidate critical steps in the conversion of biomass to methane as it delivers combined functional and phylogenetic data. Although metaproteomics analyses are challenged by sample impurities, sample complexity and redundant protein identification, and are still limited by the availability of genome sequences, recent studies have shown promising results. In the following, the workflow and potential pitfalls for metaproteomics of samples from full-scale BGP are discussed. In addition, the value of metaproteomics to contribute to the further advancement of microbial ecology is evaluated. Finally, synergistic effects expected when metaproteomics is combined with advanced imaging techniques, metagenomics, metatranscriptomics and metabolomics are addressed.

## Introduction

Over the past 10 years, conversion of biomass to methane in biogas plants (BGPs) has become a reliable source of renewable energy. In 2013, about 7500 BGPs produced 3.5% of the annual electricity demand in Germany (Fachagentur Nachwachsende Rohstoffe e.V. (FNR, 2013). In contrast to burning the biomass, the main advantage of biogas production is the possibility to utilize substrates with high water content.

In the future, the importance of biogas process might even grow, because it could be used for energy storage by biological methanation (Luo *et al*., 2012; Bensmann *et al*., 2014) or for anaerobic treatment of wastewater (Angelidaki *et al*., 2011). During the biogas process, a complex microbial community degrades biomass or organic waste including crop silage, dung, manure, sludge from wastewater treatment plants, household garbage or waste from food industry to methane. In the first step, polymeric substrates are hydrolysed to monomers by extracellular enzymes released by primary fermenters, i.e. *Clostridium thermocellum* or *Caldicellulosiruptor saccharolyticus*. Afterwards, primary fermenters such as *Clostridium acetobutylicum* convert monomers to hydrogen, carbon dioxide, short-chain fatty acids and primary alcohols. During the subsequent acetogenesis, secondary fermenters including *Syntrophomonas wolfei* metabolize primary alcohols and short-chain fatty acids to hydrogen, carbon dioxide and acetate. The released hydrogen is captured by homoacetogenic Bacteria and hydrogenotrophic methanogens such as *Acetobacterium woodii* resp. *Methanothermobacter thermoautotrophicus*. This syntrophic interaction enables the secondary fermenters to gain energy under thermodynamically unfavourable conditions. Finally, acetoclastic methanogens, i.e. *Methanosarcina barkeri*, consume acetate and convert it to methane and carbon dioxide. For a continuous high-yield biogas production, all of the metabolic pathways of these four main steps of the biogas process have to be finely tuned.

Previous attempts to optimize biogas production focused on the impact of physicochemical and technical process parameters on performance of BGPs (Appels *et al*., 2008; Ward *et al*., 2008; Holm-Nielsen *et al*., 2009; Weiland, 2010; Angelidaki *et al*., 2011). However, several problems still impair the optimal conversion of biomass to methane (Ward *et al*., 2008).

First, microbial communities degrade only 30–60% of the fed biomass (Angelidaki *et al*., 2011), because lignin and cellulose are resistant to hydrolysis. In contrast, the microbial communities in the gut of sheep (Toyoda *et al*., 2009) or termites (Burnum *et al*., 2011) are able to utilize both lignin- and cellulose-rich grass and wood with high efficiency.

Second, in order to avoid process disturbances, a lot of energy and effort is spent to adjust optimal process conditions for the microbial community, e.g. optimal ammonia concentrations (Appels *et al*., 2008) or stable process temperatures. However, microbial communities are able to adapt to challenging process conditions, partially questioning these efforts (Chen *et al*., 2008).

Third, dynamic operation of BGPs would be favourable to produce electricity on demand and to stabilize the electric grid, but is rarely applied due to risk of acidification (Munk *et al*., 2010) and missing control strategies. Dynamic operation could be applied by different feeding amounts easily, but more detailed knowledge about the metabolic limits of the microbial communities is required.

In summary, a lack of understanding concerning the composition and performance of the microbial community hinders further optimization of the biogas process (Weiland, 2010). Therefore, improvement of space–time yield of BPGs requires the clarification of the following three key questions of applied microbial ecology: (i) who is there, (ii) who is doing what with whom and (iii) how can we adjust initial conditions and control the composition of the microbial communities as previously suggested by Verstraete and colleagues (2007).

In order to answer these questions, different approaches namely microscopy, metagenomics, metatranscriptomics, metaproteomics and metabolomics are available. In particular, metaproteomics, targeting the identification of proteins/enzymes from the individual species of the microbial community, represent a promising approach. The main advantage of metaproteomics is the possibility to link the function of proteins with a certain taxonomy and to correlated their presence with metabolic activity (Wilmes and Bond, 2006). However, metaproteomics is challenged by four major problems (Muth *et al*., 2015): (i) contamination by products of biomass degradation, (ii) sample complexity, (iii) redundant protein identifications and (iv) lack of detailed databases. In this paper, the value of metaproteomics for analyses of BGPs is discussed, and an optimized workflow comprising sampling, protein purification, separation, mass spectrometry (MS), bioinformatics and result evaluation is described.

## Tools for the characterization of microbial communities

For the analysis of complex microbial communities, a wide spectrum of elaborated methods is available as shown in Table 1. Besides the characterization of genes, mRNAs, proteins and metabolites using dedicated assays, microscopic analysis of microorganisms is also a valuable option. Due to different targets, the methods provide different levels of information concerning the spatial organization, the taxonomic composition as well as the function and metabolic activity of the individual microbial species.

**Table 1:** Tools for the characterization of microbial communities

Method	Target	Spatial organization	Taxonomy	Function	Metabolic activity	Analysed parameters per run^a^	Supplements metaproteomics
microscopy	microorganisms	+	±	−	−	1 sample	indicates successful cell lysis
flow cytometry	microorganisms	−	±	−	−	1 sample/1–3 stainings	
FISH microscopy	microorganisms	+	+	±	±	1 sample/1–3 stainings	
TRFLP/DGGE	genes/mRNA	−	±	±	−	1 sample/1 gene	
TRFLP/DGGE + clone library	genes/mRNA	−	+	±	−	1 sample/1 gene	
metagenome sequencing	genes	−	+	+	−	≈10,000 contigs	database for metaproteomics
metatranscriptome sequencing	mRNA	−	+	+	±	≈10,000 contigs	database for metaproteomics
metaproteomics	proteins	−	+	+	±	≈1,000 proteins	Re-annotation of genes by proteogenomics
metabolomics	intermediates	−	−	±	+	≈10–20 intermediates	proves activity of proteins
enzyme activity assays	enzymes	−	−	±	+	1 enzyme	activity values for genes/proteins

a Numbers of analysed parameters per run are estimated. Actual numbers depend on the experimental setup.

Comparison of standard methods for the investigation of microbial communities, concerning its target, effort, price as well as the type and amount of information obtained (−, no information; ±, qualitative information; +, quantitative information). The evaluation of these methods was done to the best of our knowledge and refers to the number of analysed parameters per run. However, only a broad overview about available methods can be given within the scope of this review.

DGGE, denaturing gradient gel electrophoresis; TRFLP, terminal restriction fragment length polymorphism.

Microscopy is a well-known technology used to investigate the organization of microbial communities regarding abundance and spatial distribution (Grotenhuis *et al*., 1991). However, most microorganisms cannot be classified by morphology alone. Nevertheless, in BGPs, the F420 cofactor (Heine-Dobbernack *et al*., 1988) is involved in methanogenesis and shows an intrinsic fluorescence allowing specific detection of hydrogenotrophic methanogens. For further differentiation, specific staining methods such as fluorescence in situ hybridization (FISH) can be used (Sekiguchi *et al*., 1999; Nettmann *et al*., 2010). Nevertheless, strong background fluorescence from sample impurities, i.e. humic and fulvic acids (Senesi *et al*., 1989), often interferes with staining procedures (Hofman-Bang *et al*., 2003; Bastida *et al*., 2009). In addition to microscopy, flow cytometry can be applied to discriminate between individual strains and to follow dynamics of microbial communities (Müller *et al*., 2012). Molecular biological analysis of genes or mRNA is a more robust and precise method for the phylogenetic or functional characterization (Hofman-Bang *et al*., 2003; Klocke *et al*., 2008; Nelson *et al*., 2011; Ziganshin *et al*., 2013). In particular, the presence of 16S rRNA genes is frequently used for phylogenetic studies (Amann *et al*., 1995). The presence of functional genes or corresponding mRNA is utilized for measuring the functional diversity. Due to its low stability, the presence of mRNA is a good indicator of gene expression. While the analysis of RNA requires a previous reversed transcription to cDNA, DNA is directly amplified by polymerase chain reaction (PCR) with primers specific to phylogenetic groups or selected functional genes. Afterwards, the equally sized PCR products are separated by denaturing gradient gel electrophoresis (DGGE) or terminal restriction fragment length polymorphism (TRFL) revealing the fingerprint. In the case of 16S rRNA based community analysis, the individual microorganisms can be identified by a clone library and the actual community profile can be generated by normalization with the species-specific abundance of the 16S rRNA gene (Klappenbach *et al*., 2001). In BGPs, the functional analysis was successfully applied for quantification of the methyl CoM reductase gene and its mRNA (Munk *et al*., 2012).

The development of 454 pyrosequencing (Margulies *et al*., 2005) and illumina sequencing (Bentley *et al*., 2008) enabled the investigation of the whole metagenome/metatranscriptome of microbial communities instead of analysing single genes or individual mRNA (Schlüter *et al*., 2008; Wirth *et al*., 2012; Zakrzewski *et al*., 2012). In contrast to a metagenome, representing the genetic potential of a community, the metatranscriptome is a snapshot of the actual gene expression.

However, final metabolic activity is determined, among other factors, by the concentrations of proteins, which are strongly influenced by their half-life periods. Thus, a better description of the metabolic function of microbial communities is expected from the abundance of the microbial enzymes and proteins [metaproteome (Wilmes and Bond, 2006)]. Most proteomic approaches, however, are performed under denaturating conditions and therefore provide only information regarding the abundance of proteins instead of enzyme activities. In addition, the latter are influenced by temperature, pH value as well as on the concentration of substrates and products. Therefore, enzyme activity assays, e.g. enzymes of hydrolysis (Gasch *et al*., 2013) or enzymes of methanogenesis (Refai *et al*., 2014), were also established for analysis of microbial communities from BGPs and could be applied to confirm metaproteome data. Alternatively, concentrations of intracellular and extracellular metabolites (metabolome) could be determined as they also represent the microbial activity. The currently performed routine sampling of full-scale BGPs clearly provides only a minimum of information regarding the metabolic activity of microbial communities, e.g. the composition and amount of the substrates, the volume and composition of the gas, and the concentration of the short-chain fatty acids in the digestate (Hill and Holmberg, 1988). However, without expensive labelling of substrates with stable isotopes, metabolites cannot be assigned to phylogenetic groups.

Obviously, each method for microbial community characterization has its own advantages. Only a combination of different methods will allow to draw a more realistic picture of the microbial conversion of biomass to methane, and to derive successful measures for process design and further optimization. The problems and limits of the individual methods should be carefully considered, as done in the review of Hofman-Bang and colleagues (2003) for molecular biological methods.

## Metaproteomic workflow

**Figure 1:**
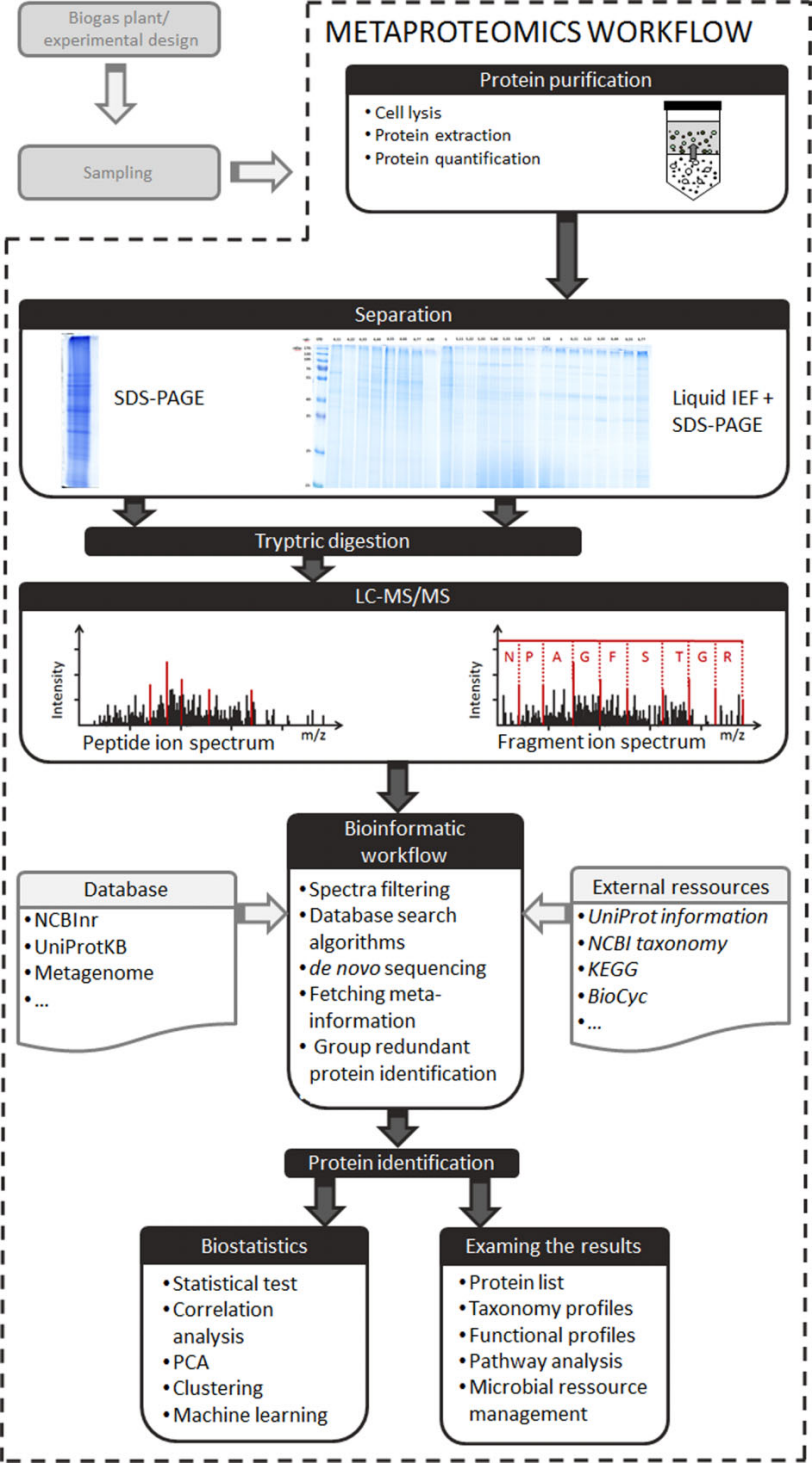
Metaproteomics workflow comprising sampling, protein purification, separation, mass spectrometry, bioinformatic workflow and result evaluation.

The implementation of metaproteomics approaches covering a wide range of BGP samples requires considering several key challenges: (i) experimental design, (ii) sampling, (iii) protein purification, (iv) protein separation, (v) liquid chromatography (LC)-tandem mass spectrometry MS/MS), (vi) bioinformatics and (vii) examining the protein identification (Figure 1).

Reproducible scientific studies require carefully planned and documented experiments. In order to correlate metaproteome data from different BGPs with process data, at least a minimum set of meta-information has to be provided, comprising BGP design, process temperature, pH value, nitrogen content, inocula, gas composition and volume as well as feed composition. In addition, technical and biological replicates are required. However, for most industrial scale BGPs, no true replicates exist, because each BGP is individual concerning its construction and operational parameters. An acceptable workaround would be to sample at least two independent technical replicates at close time points. Otherwise, investigations using lab-scale equipment are required to complement studies. Here, critical process conditions can be applied without risking the crash of a full-scale fermenter. Depending on the scientific question, simplifying the complex microbial community by feeding a synthetic medium (Wilmes *et al*., 2008; Abram *et al*., 2009) or the prior use of synthetic communities could be useful (Laube and Martin, 1981; Tatton *et al*., 1989; Scholten and Conrad, 2000; Plugge *et al*., 2010)

A key issue for metaproteome studies is sample complexity. Deeper insight into the metaproteome can be gained through a combination of orthogonal separation steps as shown by Kohrs and colleagues (2014). However, higher resolution requires significantly higher experimental effort. Researchers should consider this before initiating a comprehensive study in which insight into the metaproteome is often indispensable to validate research hypotheses. As long as a sufficient number of representative samples are retained, a more comprehensive metaproteomics analysis or the sequencing of the corresponding metagenome can be carried out.

Sampling is quite straightforward and representative as long as the BGP is well mixed, the dead volume of the sampling tube is discarded and the samples are frozen immediately. When sampling full-scale BGPs, the following issues have to be considered: (i) sampling before feeding and at same time of the day, (ii) mixing the BGP before sampling and (iii) discarding sufficient material before sampling in order to flush the sample port.

Sample preparation includes cell lysis, protein extraction, protein quantification and separation. Main problems during cell lysis and protein extraction are the high amount of sample impurities and the different levels of microbial community organization, such as scattered microorganisms, biofilms on the substrates or granules (Hofman-Bang *et al*., 2003). Consequently, robust lysis of all cells and removal of as many contaminants as possible is required. Phenol extraction followed by ammonium acetate in methanol precipitation sometimes combined with cell lysis in a ball mill has already been successfully applied to characterize samples from activated sludge (Kuhn *et al*., 2011), soil (Benndorf *et al*., 2007) and BGPs (Heyer *et al*., 2013). Phenol extraction separates proteins and humic substances and is essential when extracting proteins from full-scale BGPs. For lab-scale fermenters fed with synthetic media, cell lysis with ultrasonic sound and separation of debris from proteins by centrifugation is sufficient (Abram *et al*., 2009). Subsequent dissolution of precipitated proteins in buffers, especially after phenol extraction, is difficult but high molar urea buffers delivered good results (Keiblinger *et al*., 2012; Heyer *et al*., 2013). However, the use of high molar urea buffer or the presence of remaining humic substances may interfere with standard protein assays (Kuhn *et al*., 2011), namely Bradford (Bradford, 1976), Lowry (Lowry *et al*., 1951) and BCA (Smith *et al*., 1985) assays. In contrast, acceptable protein quantification can be achieved by using the amido black assay (Popov *et al*., 1975; Schweikl *et al*., 1989; Hanreich *et al*., 2013) or by quantification of protein intensities in sodium dodecyl sulfate polyacrylamide gel electrophoresis (SDS-PAGE).

Due to the high sample complexity, the proteins have to be separated prior to MS analysis. Common approaches are protein separation according to molecular weight or isoelectric point, e.g. SDS-PAGE (Laemmli, 1970) or two-dimensional polyacrylamide gel electrophoresis (2D-PAGE) (Klose, 1975; O’Farrell, 1975). Subsequent protein fractions are tryptically digested in-gel into peptides (Shevchenko *et al*., 1996). A complete gel-free approach involving separation of tryptic peptides by one or higher dimensional LC appears feasible (Link *et al*., 1999; Washburn *et al*., 2001; Wolters *et al*., 2009). However, running the proteins through a SDS-gel without separation and subsequent in-gel digest is useful because sample impurities remain in the gels. Furthermore, such a step prevents clogging of the columns and capillaries of the chromatographic system (Kohrs *et al*., 2014). For routinely metaproteomics, SDS-PAGE procedures achieved best results since remaining impurities from environmental samples seem to hinder reproducible separation of proteins by 2D-PAGE. For improving resolution of metaproteomics, liquid-isoelectric focusing (IEF) can be carried out prior to SDS-PAGE (Kohrs *et al*., 2014). Alternatively, ultracentrifugation can be used to separate the cellular and the extracellular fractions of proteins (Binner *et al*., 2011).

MS, often combined with LC, is the standard approach for protein identification. In MS, peptides are ionized and separated according to their mass-to-charge ratio. In order to distinguish between peptides with identical amino acid composition but different amino acid order, peptide ions are further fragmented. For protein identification, these fragment spectra are compared against a protein database. A complete overview about MS techniques can be found in Wöhlbrand and colleagues (2013).

For samples with high complexity containing a large number of peptides, separation of peptides by LC and high-resolution MS are essential. Nevertheless, the probability that peptides with similar mass-to-charge ratio coelute from the LC systems increases drastically. Finally, the common fragmentation results in low-quality spectra that fail in a database search. Worse, the number of peptides with different mass-to-charge ratios is so high that, due to the limited scan and separation speed of the MS, only 5–30 of the most abundant peptide ions can be analysed in one cycle. Although certain rules are applied to carefully select peptides for fragmentation, this selection is still often random due to the high number of peptides. Therefore, the reproducibility between such LC-MS/MS experiments is low (Tabb *et al*., 2010). Running technical replicates in LC-MS/MS and extending the LC gradients are appropriate strategies to manage this problem. Besides protein identification, quantification of at least key proteins is often important for the characterization of microbial communities. Common quantification strategies include chemical labelling, isotopic labelling, label-free quantification as well as quantification of protein intensity in gels. Signals from fluorescence labels, often used in gel-based approaches, can be disturbed by intrinsic fluorescence of humic-like sample impurities (Li *et al*., 2004). In addition, the remaining humic compounds can also react with established chemical labels (Gygi *et al*., 1999; Lottspeich and Kellermann, 2011). Due to this uncertainty, label-free quantification by peptide respective spectra abundance remains the last option. However, normalization of abundances is beneficial and the exponential modified protein abundance index (Ishihama *et al*., 2005) or the normalized spectra abundance factor (Zybailov *et al*., 2007) are frequently applied. Another promising quantification approach is the metabolic labelling with isotopically labelled substrates (Jehmlich *et al*., 2008). Incorporation of stable isotopes into proteins can be monitored by MS and allows to draw conclusions about metabolic activity in the microbial community. However, the application is restricted to microcosm experiments due to the high costs for fully labelled substrates.

Routinely, peptide and protein identification are carried out by comparison of fragment spectra against theoretical spectra from a database by algorithms, e.g. Mascot (Perkins *et al*., 1999) or X!Tandem (Craig and Beavis, 2004). Standard databases for protein identification are NCBInr (Acland *et al*., 2014), UniProtKB/Swiss-Prot or UniProtKB/TrEMBL (Consortium, 2012). With respect to metaproteomics, more specific databases or searches against metagenomes from the same or similar samples [e.g. for BGP samples (Schlüter *et al*., 2008; Rademacher *et al*., 2012; Wirth *et al*., 2012; Zakrzewski *et al*., 2012)] resulted in the identification of more proteins and are strongly recommended.

Raw data contains many low-quality spectra (Muth *et al*., 2015). During preprocessing, low-quality spectra can be removed without any significant loss of information (Ma *et al*., 2011). Recently, preprocessing and data handling was embedded into complete bioinformatic platforms, e.g. OpenMS (Sturm *et al*., 2008), Proteome Discoverer or ProteinScape (Thiele *et al*., 2010).

Besides a probability-based score as a measure for correctness of peptide identification, the false discovery rate (FDR) evolved in the proteomic community as a standard (Elias *et al*., 2005). FDR is mainly influenced by database size, e.g. a doubling of the database size doubles more or less the probability of false positive hits and thus doubles the FDR. Therefore, searching against large databases can cause the removal of valuable hits in order to reach a low FDR (e.g. less than 5%).

The problem of metaproteomics in contrast to other proteomics with pure or defined mixed cultures is that the taxonomic composition of complex microbial communities is not known and the database cannot be reduced to keep the number of false positive hits low. In this context, the idea of Jagtap and colleagues (2013) to repeat the search in a qualified database with reduced size containing only sequences from species identified in a first search round seems to be an option. The number of false positive hits decreases and consequently more spectra are regarded as correctly identified. However, this strategy may lead to an underestimation of the FDR and mask the lack of suitable database entries for many microorganisms due to cultivation problems (Amann *et al*., 1995).

In the next step, protein identification is achieved based on identified peptides (Bradshaw *et al*., 2006). Although identifications based on two peptide per protein are favoured in peer-reviewed journals, the so-called ‘single hit wonders’ are not necessarily worse. In fact, identifications based on single peptides are also accepted when using high-resolution MS because the quality of the peptide identification is also considered important (Gupta and Pevzner, 2009). Nevertheless, reliability and number of correctly identified peptides and proteins can be increased by combining multiple algorithms (Ma *et al*., 2011; Vaudel *et al*., 2011). Even the best algorithm can only identify proteins whose sequence is covered in a database. An approach to overcome this pitfall is *de novo* sequencing of peptides using acquired spectra (Frank and Pevzner, 2005), and to search for homologue proteins using the MS-driven basic local alignment search tool (MS-BLAST) (Shevchenko *et al*., 2001). However, the evaluation of *de novo* results requires manual inspection. Therefore, a more straightforward strategy is sequencing the metagenome of the analysed sample.

After successful protein identification, the importance of a single identification can be improved by acquiring meta-information concerning taxonomy and function from repositories, e.g. UniProt (Consortium, 2012). Moreover, redundant protein identifications due to similar peptides from homologue proteins can be grouped based on a similar peptide sets (Schneider *et al*., 2012), one shared peptide (Kohrs *et al*., 2014; Lü *et al*., 2014) or by sequence similarity [e.g. UniRef-Cluster (Suzek *et al*., 2007)] to so called metaproteins (Muth *et al*., 2015). Finally, protein taxonomy can be redefined by the common ancestor taxonomy of all proteins in a group (Huson *et al*., 2007). It allows a reliable phylogenetic assignment of metaproteins avoiding risky assignments on species or strains.

**Figure 2:**
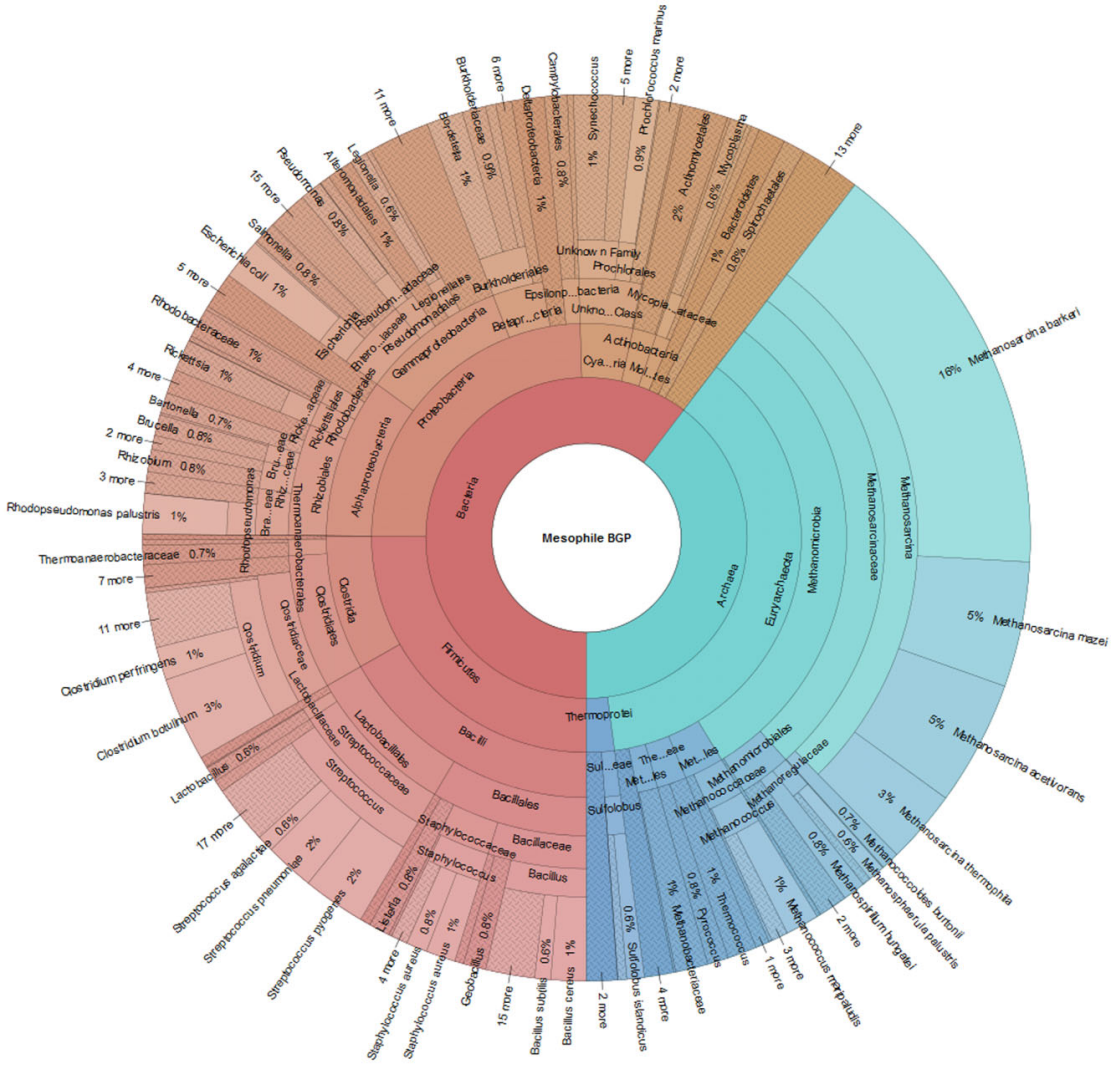
Krona plot of a mesophilic BGP, based on the data of Kohrs and colleagues (2014). The abundance of the taxonomic groups corresponds to the percentage of spectra based on a total number of 9485 spectra.

For a better survey, taxonomic composition can be visualized in a Krona plot (e.g. Krona plot for a mesophilic/thermophilic BGP: Figure 2; Fig. S1) (Ondov *et al*., 2011) that is based on identified peptides or spectra, and National Center for Biotechnology Information taxonomy (Acland *et al*., 2014). For comparison of taxonomic profiles from different samples or time points, the richness of species, their community organization and their dynamics can be calculated, as extensively discussed in the concept of Microbial Resource Management (Verstraete *et al*., 2007; Marzorati *et al*., 2008; Wittebolle *et al*., 2009).

**Figure 3:**
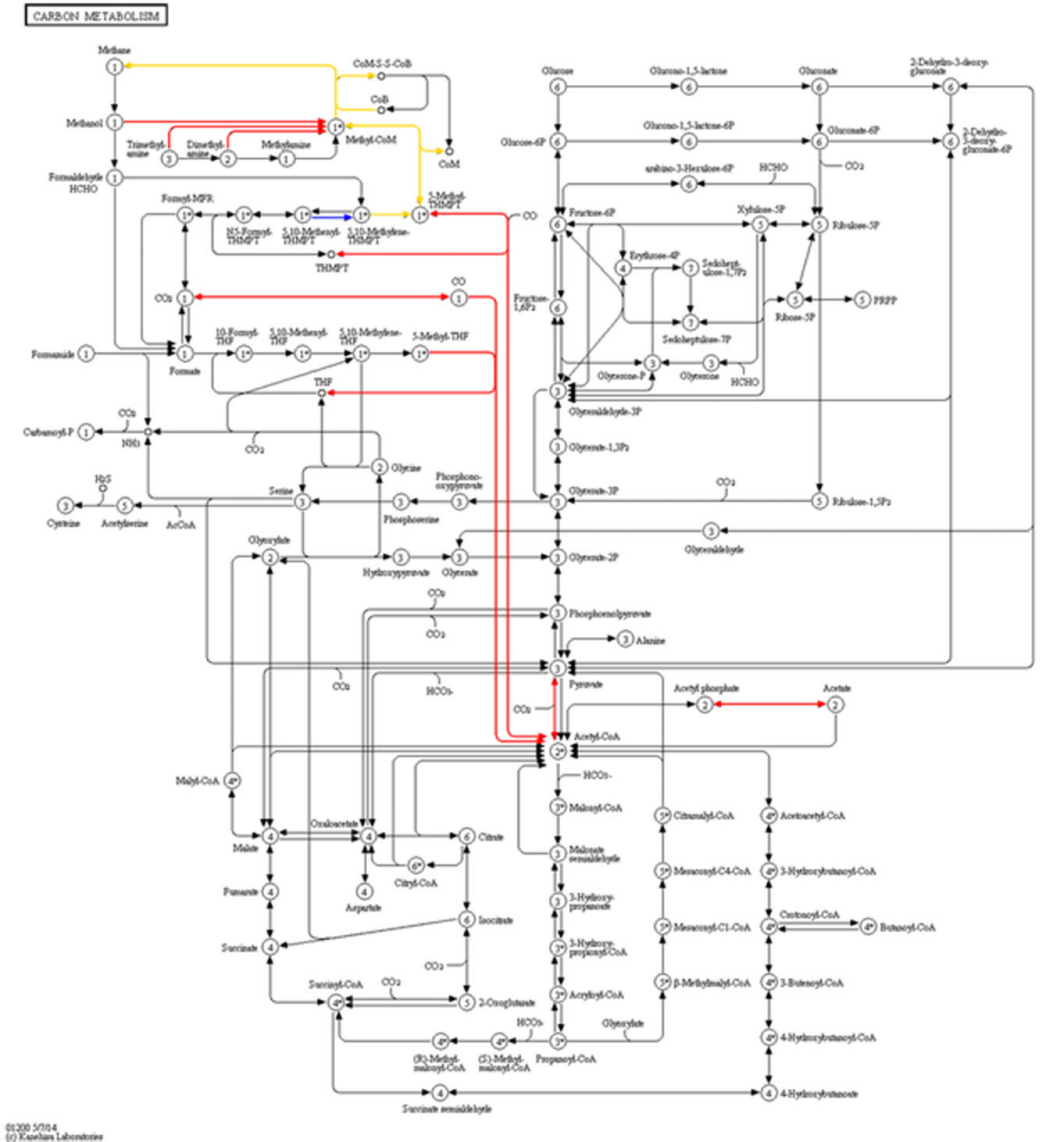
Carbon metabolism of a mesophilic biogas plant, based on the data of Kohrs and colleagues (2014). KEGG pathway map of the carbon metabolism with the identified proteins for methanogenesis from different Archaea (red: *Methanosarcinales*, blue: *Methanomicrobiales*, gold: both groups).

Shifting to protein functions, overview plots, such as a Voronoi Treemap (Bernhardt *et al*., 2013) or a common pie chart, based on gene ontologies (Ashburner *et al*., 2000) or UniProt Keywords (Consortium, 2012) are beneficial. Even more important is the assignment of identified proteins to biochemical pathways. A straightforward mapping to MetaCyc pathways (Caspi *et al*., 2014) or Kyoto Encyclopedia of Genes and Genomes (KEGG) pathways (Figure 3, Fig. S2) (Kanehisa and Goto, 2000) can be achieved using KEGG ontologies or enzyme commission numbers (Bairoch, 2000). Often, proteome studies result in long lists of upregulated and downregulated proteins confirmed by statistical tests (Karp and Lilley, 2007). For better exploitation of data, correlation analysis between taxa, functions or process parameters can reveal unexpected functional relationships improving the knowledge about the microbial community. Moreover, differences between BGPs can be monitored by principal component analysis or cluster analysis of protein or taxonomic profiles, e.g. cluster of different BGPs based on SDS-PAGE profiles (Heyer *et al*., 2013). In the future, the use of machine learning algorithms (Kelchtermans *et al*., 2014) might result in further improvements.

In microbial ecology, a wide mixture of different methods are commonly applied to investigate a specific problem. Hence, knowledge of how to combine these methods is important. First of all, a standardization of sample preparation is essential. With regard to the use of multi-omic approaches, the sample preparation workflow should comprise most analytes namely DNA, RNA, proteins and metabolites in an adequate manner (Roume *et al*., 2013). As previously discussed in *Tools for the characterization of microbial communities* (Table 1), metaproteomics delivers thorough information about taxonomy and function while conclusions regarding community organization and metabolic activity can only be obtained to a limited degree. Further insight in microbial communities could be gained by combining data from advanced microscopy approaches, e.g. FISH and metaproteomics. For instance, hypotheses regarding syntrophic interaction of *Coprothermobacter* and *Methanothermobacter* in a thermophilic reactor treating thermally pretreated sludge (Gagliano *et al*., 2014) might have benefited from an additional proteomic study. Moreover, protein identification in metaproteomics profits from high-quality genome databases, making metagenomics/metatranscriptomics and metaproteomics partners rather than competitors. The fact that proteomics can also be used to improve the quality of gene annotation in genome studies (Gupta *et al*., 2007) indicates that this interaction might not be an ‘one way road’. In particular, ‘proteogenomics’ approaches might be applied to assist the annotation of metagenome data using the recently published proteogenomic software Peppy (Risk *et al*., 2013). Another option might be the combination of flow cytometry and metaproteomics (Jehmlich *et al*., 2010) as cell sorting enriches microbial subpopulations and therefore reduces complexity of samples prior to metaproteome analysis.

## Advances in the field enabled by metaproteomics

So far, only a few metaproteome studies of BGPs were carried out (Table 2) (Abram *et al*., 2009; 2011; Hanreich *et al*., 2012; 2013; Yan *et al*., 2012; Heyer *et al*., 2013; Kohrs *et al*., 2014; Lü *et al*., 2014). Of those, only the work of Hanreich and colleagues (2012), Heyer and colleagues (2013) and Kohrs and colleagues (2014) analysed full-scale BGPs. Most studies reported on the massive problems related to sample impurities, especially humic-like substances, requiring extensive sample preparation with phenol. As a consequence, the separation of proteins by 2D-PAGE, even with improvement by paper bridge loading (Hanreich *et al*., 2012), sometimes failed (data not shown). In order to reduce the high sample complexity, proteins can be separated by SDS-PAGE as discussed in Heyer and colleagues (2013) and Kohrs and colleagues (2014). In addition to SDS-PAGE, Lü and colleagues (2014) used IEF to separate the proteins.

**Table 2:** Overview about previous metaproteome studies

Author	Fermenter	Substrate	Process temperature	Separation method	Identified proteins
Abram *et al*. (2011)	3–5 L lab scale	synthetic glucose-based wastewater	15°C	2D-PAGE (388 spots)	33 proteins
Yan *et al*. (2012)	2 L lab scale	blue algae, sludge	35°C	2D-PAGE (200–300 spots)	3 proteins
Hanreich *et al*. (2012)	8 L lab scale	beet silage, chopped rye	55°C	2D-PAGE (350 spots)	7 enzymes of methanogenesis + several housekeeping proteins
Hanreich *et al*. (2013)	500 ml batch test	straw, hay, digestate from maize fermentation	38°C	2D-PAGE (300 spots)	80 proteins
Heyer *et al*. (2013)	6 agricultural biogas plants	mainly grain silage, slurry and manure	5× mesophilic 1× thermophilic	SDS-PAGE	100–150 proteins
Kohrs *et al*. (2014)	mesophilic agricultural biogas plants	maize silage, forage rye, cattle manure and slurry	43°C	LC-MS/MS, SDS-PAGE, Liquid-IEF + SDS-PAGE	757–1,639 proteins
	thermophilic agricultural biogas plants	maize whole crop silage and poultry manure	52°C	LC-MS/MS, SDS-PAGE, Liquid-IEF + SDS-PAGE	1,663–2,091 proteins
Lü *et al*. (2014)	1 L bottle	office paper + sludge + buffer	55°C	LC-MS/MS, SDS-PAGE, Liquid-IEF	500 proteins

While in early metaproteome studies, only a few proteins were detected (Abram *et al*., 2009; 2011; Hanreich *et al*., 2012; 2013; Yan *et al*., 2012), recent high-resolution separations using liquid IEF and SDS-PAGE (Kohrs *et al*., 2014; Lü *et al*., 2014) enabled the identification of up to 1000 proteins (Table 2). Assignment of these 1000 protein identifications to the biogas process enabled to cover the main steps of hydrolysis, fermentation, acetogenesis and methanogenesis. In addition, the most important phylogenetic groups known to be involved in biomass conversion to methane were identified.

The majority of the identified bacterial proteins belonged to the orders *Actinobacter*, *Bacteriodia*, *Bacilli*, *Chlostridiales*, *Thermotogae* and different *Proteobacter* groups. Archaeal proteins were dominated by the orders *Methanobacteria* and *Methanomicrobia*. A comparison of phylogenetic profiles derived from metaproteomics and molecular biological studies revealed significant differences in the relative abundance of methanogens [about 20–30% in metaproteome data compared with the 4% derived by metagenomics (Hanreich *et al*., 2013)]. Both approaches may be subject to bias resulting from differences in cell lysis and extraction of proteins respective genes. When comparing both results with predicted community structures based on modelling of the biogas process, e.g. the Anaerobic Digestion Model number 1 (Batstone *et al*., 2002), abundance of methanogens based on metaproteome data seems to be more correct. Moreover, the difference between abundances based on metaproteome and genomic data is not restricted to methanogens. For example, Lü and colleagues (2014) were astonished about only a few proteins from the genus *Gelria* by metaproteomics, although it was highly abundant in the pyrosequencing data. In this case, the bias might have been introduced by the lack of protein entries for *Gelria* in the UniProt database.

Besides proteins from Archaea and Bacteria, several proteins from plants and animals are frequently identified in samples from BGPs. They are originated from plant feedstock or manure and represent the incomplete usage of substrate. In addition, a few proteins were identified to belong to Fungi (Kohrs *et al*., 2014) and to Bacteriophages (Lü *et al*., 2014). Most likely, the identified proteins were not correctly assigned phylogenetically due to homologous protein sequences. At present, however, it cannot be ruled out completely that Fungi (Trinci *et al*., 1994) or Bacteriophages (Suttle, 2007) have any relevance in BGPs.

As already discussed, the main advantage of metaproteomics is the functional characterization of microbial communities together with the phylogenetic assignment. Lü and colleagues (2014) showed, for example, that hemicellulose was hydrolysed by the genus *Caldicellulosiruptor* and that celluloses were degraded by the cellulosome of *Clostridium thermocellum*. Surprisingly, Lü and colleagues (2014) also observed a high proteolytic activity from *Clostridium proteolyticus*, indicating its function as predator or scavenger of dead biomass. The observed proteolytic activity nicely confirmed a study (Binner *et al*., 2011) demonstrating the fast degradation of externally added cellulolytic enzymes.

For the subsequent fermentation step, mainly proteins from sugar uptake, glycolysis and to some extent from the pentose phosphate pathway were identified. This is in accordance with carbohydrates as the major substrate of biogas production. Whether the Entner–Doudoroff pathway is of relevance for fermentation (Abram *et al*., 2011) or not (Abram *et al*., 2011; Lü *et al*., 2014) depends on process conditions. Pyruvate, which is produced during the glycolysis, is further converted to ethanol, acetate, lactate (Kohrs *et al*., 2014) respectively to propionate, butyrate and butanoate (Lü *et al*., 2014).

Amino acids derived from feedstock proteins are fermentable substrates and precursors for synthesis of microbial biomass. Based on metaproteome data, Heyer and colleagues (2013) reported about an imbalance of amino acids available for microbial anabolism. On the one hand, the enzyme glutamate dehydrogenase degrades glutamate and represents catabolism; on the other hand, enzymes like aspartokinase and dihydrodipicolinate reductase represent anabolism and are involved in *de novo* synthesis of methionine, lysine and threonine surpassing their low proportion in maize protein (Ridley *et al*., 2002).

In contrast to the previous steps of the biogas process, the investigation of acetogenesis is even more challenging. In fact, Lü and colleagues (2014) were able to identify the majority of key bacterial enzymes for the acetyl–CoA pathway. However, this pathway not only enables the production of acetate from hydrogen and carbon dioxide but can also be used to oxidize acetate to carbon dioxide. The impact of these two possibilities on the biogas process is further discussed in the review of Müller and colleagues (2013). Consequently, the direction of this pathway can only be determined based on the presence of species identified by metaproteomics or the absence of proteins from acetoclastic methanogenesis.

Proteins involved in methanogenesis are highly abundant in metaproteomes. Nearly all enzymes of the hydrogenotrophic and the acetoclastic pathway were identified. Under psychrophilic and mesophilic conditions, the acetoclastic pathway is favoured (Abram *et al*., 2011; Hanreich *et al*., 2013; Heyer *et al*., 2013; Kohrs *et al*., 2014), whereas, under thermophilic conditions, the hydrogenotrophic pathway is preferred (Kohrs *et al*., 2014; Lü *et al*., 2014). The presence of the acetoclastic pathway under thermophilic conditions was only identified once (Hanreich *et al*., 2012), and seems to be an individual case. Furthermore, enzymes for methanogenesis from single carbon atom compounds were detected (Heyer *et al*., 2013; Kohrs *et al*., 2014) demonstrating the usage of methanol and methylamines released from biomass in BGPs.

Although MS-based metaproteomes of different samples show some similarities due to high abundance of methanogenic enzymes and also the presence of similar dominating phylogenetic groups, each BGP seems to have its own protein signature. Surprisingly, separation of proteins by SDS-PAGE is sufficient to produce individual protein patterns (Heyer *et al*., 2013). These protein patterns were stable for time periods of several months and changes were correlated to process disturbance, namely an acidification of the BGP. Subsequent protein identification revealed a drastic decrease of the concentration of the enzyme methyl CoM reductase in advance of acidification. Accordingly, this key enzyme of methanogenesis could be used as a predictive biomarker. A low level of the corresponding mRNA (from mcrA gene) was previously reported to be correlated to disturbed methanogenesis (Munk *et al*., 2012).

## Conclusion and outlook

In depth, analysis of microbial communities in BGPs is required to use their full potential for biogas production. The comparison of methods for the characterization of microbial communities and recent results regarding the functional and taxonomic composition of these microbial communities obtained by various research groups showed that metaproteomics is developing into a powerful tool for the exploration of the biogas process. Besides identifying major pathways of biomass degradation, it links single metabolic pathways with microbial taxa. Each BGP shows its own, time stable protein pattern. Strong alterations in this pattern can be linked with process disturbances, and some enzymes were identified as potential biomarkers for process monitoring and fault detection.

Metaproteome analysis of BGPs is still hampered, however, by sample impurities, sample complexity, redundancy of protein identifications and a lack of genome sequences required for protein identifications. Nevertheless, the presented workflow overcomes at least parts of these problems. In the future key issues to be addressed include comprehensive sample preparation, a suitable protein separation, grouping of redundant proteins and the incorporation of meta-information from online repositories. In particular, more efficient protein extraction, improved MS and new algorithms for the verification of protein identification are urgently required to further improve this workflow and to exploit the full potential of metaproteomics. In addition, it has to be taken into account that metaproteomics is no stand-alone approach. For comprehensive analysis of microbial communities, metaproteomics should be applied in concert with microscopy, cytometry, metagenomics, metatranscriptomics and metabolomics.

## Supporting Information

**Fig. S1.** Krona plot of a thermophilic BGP, based on the data of Kohrs and colleagues (2014) for a thermophilic BGP. The abundance of the taxonomic groups corresponds to the percentage of spectra based on a total number of 18139 spectra. **Fig. S2.** Carbon metabolism of a thermophilic BGP, based on the data of Kohrs and colleagues (2014). KEGG pathway map of the carbon metabolism with the identified proteins for methanogenesis from different Archaea (red: *Methanosarcinales*, blue: *Methanomicrobiales*, green: proteins of anabolism).
