# Supplementary figures and images for: Metaproteomics of complex microbial communities in biogas plants
Source: Microb Biotechnol. 2015 Apr 15;8(5):749–63. doi: 10.1111/1751-7915.12276 (PMC4554464; doi:10.1111/1751-7915.12276)

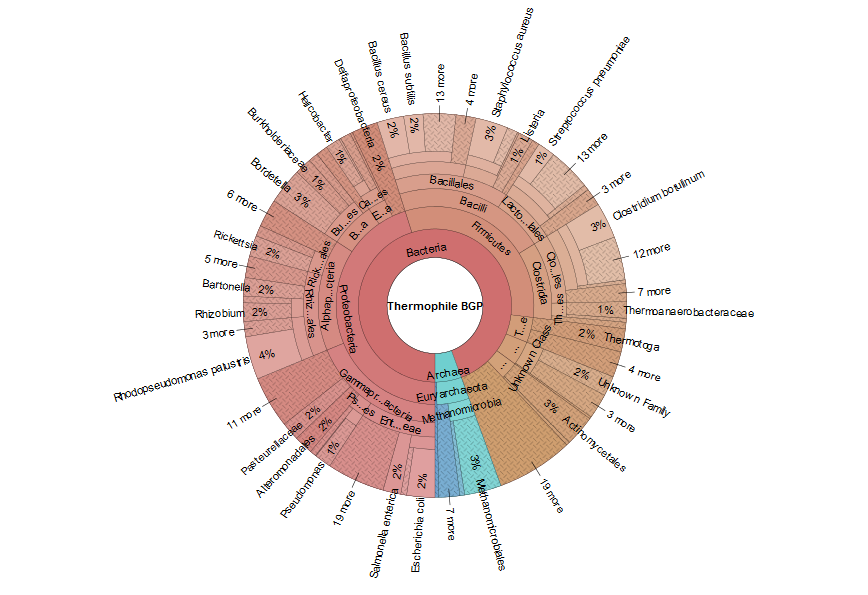

Supplement: Supplementary file 1 [file mbt20008-0749-sd1.zip › MBT2_12276-supp-0001-Supplemetary_Figure_1.tif]

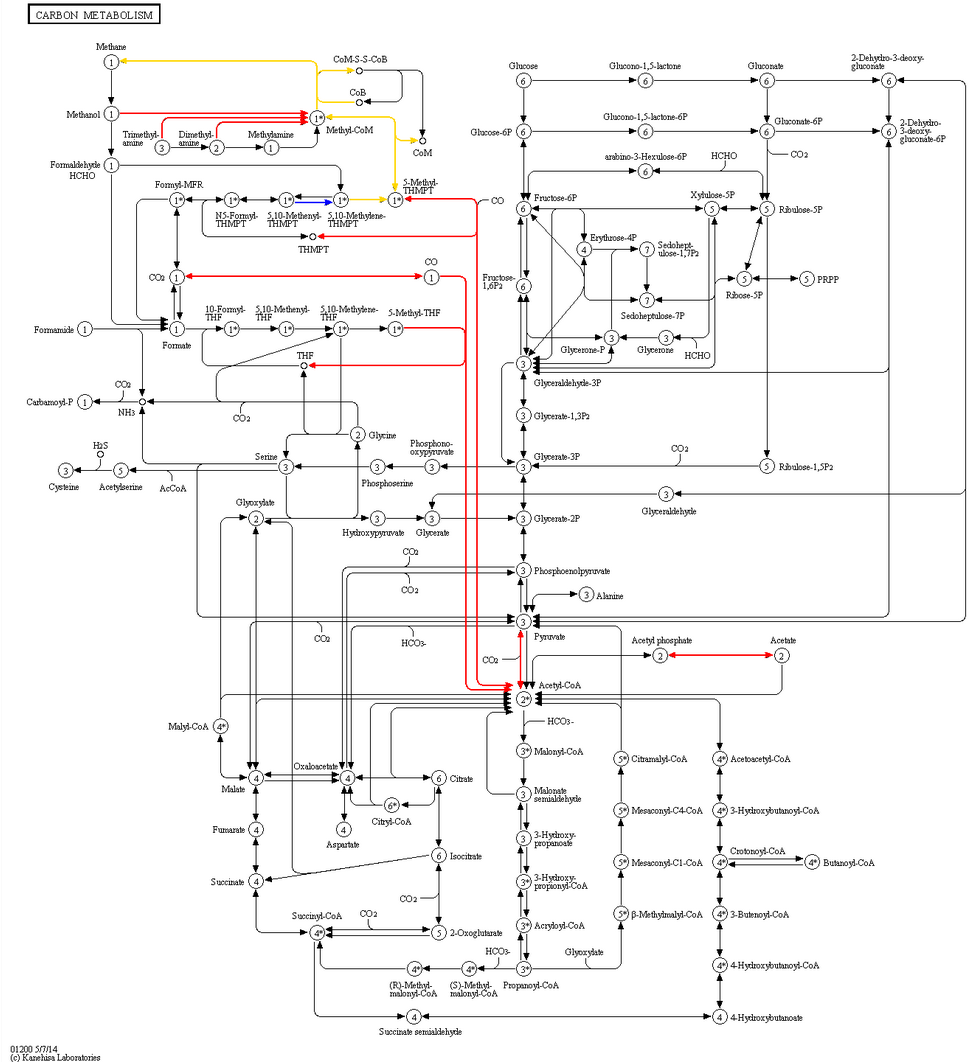

Supplement: Supplementary file 1 [file mbt20008-0749-sd1.zip › MBT2_12276-supp-0002-Supplementary_Figure_2.tif]
